# Analysis of Genetic Diversity and Population Structure of Sesame Accessions from Africa and Asia as Major Centers of Its Cultivation

**DOI:** 10.3390/genes7040014

**Published:** 2016-04-12

**Authors:** Komivi Dossa, Xin Wei, Yanxin Zhang, Daniel Fonceka, Wenjuan Yang, Diaga Diouf, Boshou Liao, Ndiaga Cissé, Xiurong Zhang

**Affiliations:** 1 Key Laboratory of Biology and Genetic Improvement of Oil Crops, Ministry of Agriculture, Oil Crops Research Institute of the Chinese Academy of Agricultural Sciences, No. 2 Xudong 2nd Road, 430062 Wuhan, Hubei, China; dossakomivi@gmail.com (K.D.); weixin@caas.cn (X.W.); zhangyanxin@caas.cn (Y.Z.); yangwenjuanqau@163.com (W.Y.); lboshou@hotmail.com (B.L.); 2 Centre d’Etudes Régional pour l’Amélioration de l’Adaptation à la Sécheresse (CERAAS), BP 3320 Route de Khombole, Thiès 21000, Senegal; daniel.fonceka@cirad.fr; 3 Centre de coopération internationale en recherche agronomique pour le développement (CIRAD), UMR AGAP, F-34398 Montpellier, France; 4 Laboratoire Campus de Biotechnologies Végétales, Département de Biologie Végétale, Faculté des Sciences et Techniques, Université Cheikh Anta Diop, BP 5005 Dakar-Fann, Dakar 107000, Senegal; diaga.diouf@ucad.edu.sn

**Keywords:** *Sesamum indicum*, genetic diversity, geographic origin, Asia, Africa, SSR

## Abstract

Sesame is an important oil crop widely cultivated in Africa and Asia. Understanding the genetic diversity of accessions from these continents is critical to designing breeding methods and for additional collection of sesame germplasm. To determine the genetic diversity in relation to geographical regions, 96 sesame accessions collected from 22 countries distributed over six geographic regions in Africa and Asia were genotyped using 33 polymorphic SSR markers. Large genetic variability was found within the germplasm collection. The total number of alleles was 137, averaging 4.15 alleles per locus. The accessions from Asia displayed more diversity than those from Africa. Accessions from Southern Asia (SAs), Eastern Asia (EAs), and Western Africa (WAf) were highly diversified, while those from Western Asia (WAs), Northern Africa (NAf), and Southeastern Africa (SAf) had the lowest diversity. The analysis of molecular variance revealed that more than 44% of the genetic variance was due to diversity among geographic regions. Five subpopulations, including three in Asia and two in Africa, were cross-identified through phylogenetic, PCA, and STRUCTURE analyses. Most accessions clustered in the same population based on their geographical origins. Our results provide technical guidance for efficient management of sesame genetic resources in breeding programs and further collection of sesame germplasm from these different regions.

## 1. Introduction

Sesame (*Sesamum indicum* L.) has been described as one of the oldest oilseed plants used by humans [1,2]. It is a diploid species with 2*n* = 2x = 26 chromosomes that belongs to the family of Pedaliaceae, *Sesamum* genus, and is the most commonly cultivated edible oil crop species out of over 30 species in this genus [3,4]. It is predominantly considered as a self-pollinated plant, although low percentage of cross pollination is reported [5].

Sesame seeds have the highest oil content (~55%) among major oilseeds [6]. Beyond the traditional use for direct consumption, sesame seeds are also used as an active ingredient in antiseptics, bactericides, viricides, disinfectants, moth repellants, and antitubercular agents because they contain natural antioxidants such as sesamin, sesamol, and sesamolin [7,8]. The potentially beneficial effects of sesame on human health have recently renewed interest in this ancient crop [9].

Africa and Asia account for more than 96% of the world sesame seed production, with India, Sudan, and China as leading producer countries [10]. Sesame is thus a major crop in these continents, with a large number of varieties [11,12,13,14].

The origin of cultivated sesame and the process underlying its domestication have been controversial, though Africa and Asia were identified as probable origins [3,15,16,17,18,19,20]. Later evidences from inter-specific hybridization, molecular, and lignans analyses suggested the Indian subcontinent as the center of domestication and the probable wild progenitor is believed to be *Sesamum malabaricum* Burm [13,21].

Genetic diversity of crops plays an important role in sustainable development and food security [22], as it allows adaption to various biotic and abiotic stresses. The African and Asian continents have been projected to be the hardest hit by the negative effects of climate change on crop production [23], which will result in large parts of these continents becoming drier and/or suffering floods. Currently, breeding efforts in sesame have been aimed at the production of white seeded varieties with high market value, shorter duration, and inbuilt tolerance to drought and high temperatures. In addition, attributes such as resistance to phyllody, higher yields, improved plant architecture, and indehiscent capsules have also been incorporated [14].

Knowledge of the genetic diversity and population structure of germplasm collections is an important foundation for crop improvement and a key component of effective conservation and breeding strategies [24]. It is therefore important to assess in detail the genetic diversity of large accessions of cultivated sesame in these continents for proper and efficient utilization in breeding and also to make inferences about the history of its domestication.

Molecular characterization is now the favorite way to quantify variation within germplasm samples. Many studies have dissected the genetic diversity of sesame accessions using different types of molecular markers (Random Amplified Polymorphic DNA (RAPD) [25,26,27], Inter-simple Sequence Repeats (ISSR) [28], Amplified Fragment Length Polymorphism (AFLP) [9], Sequence-Related Amplified Polymorphism (SRAP) [29], Expressed Sequence Tags-SSR (EST-SSR) [30], Simple Sequence Repeats (SSR) [31,32,33], or a combination of molecular markers [29,34,35]. However, few studies have been conducted by using large samples of sesame accessions from Africa and Asia, which are the major centers of sesame cultivation. There, is therefore, the need to conduct large-scale genetic diversity analysis among the accessions of sesame from these two continents that have been frequently involved in the exchange of germplasm.

In this study, we examined the genetic diversity of a representative set of sesame accessions from 22 countries of Africa and Asia. The overall goal is to provide a comprehensive insight into sesame diversity between and within these continents for a better management of the genetic resources used in breeding programs.

## 2. Experimental Section

### 2.1. Plant Materials

A total of 96 accessions of cultivated sesame (*Sesamum indicum* L., 2*n* = 2x = 26), comprised of landraces and modern cultivars representing geographically and phenotypically wide variation, were used in this study. Among these accessions, 48 were collected in nine African countries and 48 in 13 Asian countries (see Table 1 and Table S1 in the Supplementary materials). All samples from Asia and some samples from Africa were obtained from a large collection of sesame accessions preserved at the China National Genebank, Oilcrops Research Institute, Chinese Academy of Agricultural Sciences. These samples have been purified through self-pollination for generations. The rest of the accessions were personally collected by the first author. The field experiment was conducted at the Oilcrops Research Institute located in Wuhan, Hubei province (China).

### 2.2. DNA Extraction

Leaves from 10 bulked three-week-old seedlings per accession were used for DNA isolation according to the Cetyltrimethyl Ammonium Bromide (CTAB) method [34]. Moreover, to reveal the extent of the intra-accession variability of some accessions from West Africa, 288 individuals from 24 accessions were analyzed using a single plant DNA extraction approach. DNA quality and quantity were assessed by spectrophotometry (NanoDrop 2000, Thermo Scientific, Wilmington, DE, USA). DNA samples were stored at −20 °C, for further use.

### 2.3. PCR and Electrophoresis

**Figure 1:**
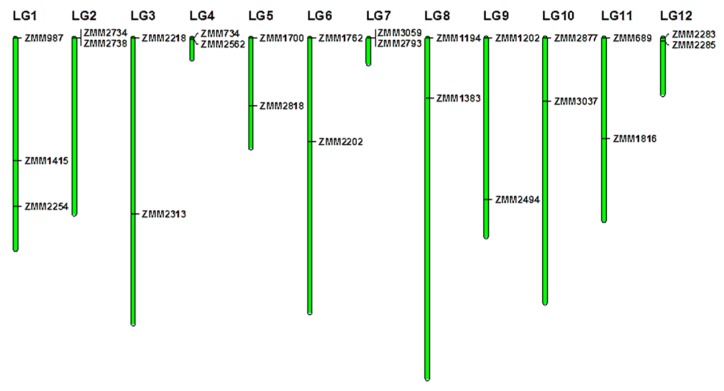
Sesame genome-wide distribution of the 33 polymorphic markers used in this study. LG1–LG16 represent the identified Linkage Groups of the sesame genome [36]. Green bars represent linkage groups. Black lines indicate the locations of primers on linkage groups.

SSR polymorphism screening was first performed using 4000 candidate markers and six accessions (three accessions from Africa and three from Asia). A total of 33 highly polymorphic SSR markers [33] providing coverage across all the 16 Linkage Groups (LG) reported in the first draft of the sesame genome [36] were selected to scan for polymorphism between the accessions (Figure 1, Table S2). Polymerase Chain Reaction (PCR) with the SSR primers was performed in a total volume of 15 μL containing 30 ng of DNA, 1 pmol of each primer, 0.2U Taq DNA polymerase, and 2x Reaction Mix (Tiangen Biotech, Beijing, China) supplied together with the dNTPs and MgCl_2_. All PCRs were conducted in 96-well plates in a S-1000 Thermal Cycler (Bio-Rad, Hercules, CA, USA). The PCR cycles were 94 °C (5 min), 35 cycles of 94 °C (30 s), 55 °C (30 s), 72 °C (30 s), followed by the extension step for 5 min at 72 °C. The amplified products were separated in 6% denaturing polyacrylamide gel and visualized by silver staining as described by [34].

### 2.4. Scoring and Data Analysis

For each locus across the genotypes, allele scoring was done manually based on the presence of a particular size allele in each of the germplasm samples. Presence was denoted as “1” and absence of an allele as “0.” For variability analysis within accessions, only two individuals from the same accession (K1712) displayed different alleles from the 10 others at one marker. Thus, further analyses focused on the bulked samples of the 96 accessions, which were grouped into six populations according to their geographical origins as indicated in Table 1. The number of alleles (Na), Effective number of alleles (Ne), Nei’s Gene Diversity (He), Observed heterozygosity (Ho), and Shannon’s Information Index (I) were estimated using POPGENE version 1.32 (University of Alberta, Edmonton, Canada) [37]. Major Allele Frequency (MAF), Number of private alleles (Np), and Polymorphic Information Content (PIC) were calculated with the software PowerMarker version 3.25 (NC State University, Raleigh, NC, USA) [38]. In addition, Analysis of Molecular Variance (AMOVA) was done using GENALEX 6.4 (The Australian National University, Canberra, Australia) [39], in order to estimate the genetic structure between and among geographical regions and continents. Since sesame populations from North Africa, West Asia, and Southeast Africa have very low sample sizes compared to other populations, they were not considered in the AMOVA analysis for geographical regions in order to avoid bias in the analyses. However, all accessions, according to their continent of origin (Asia or Africa), were included in the AMOVA analysis for continents. The significance of variance components was tested by permuting the DNA marker data 999 times. The 33 markers used in this study were mapped onto the 16 Linkage Groups (LGs) of sesame genome according to their physical positions using MapChart 2.3 (Wageningen UR, Wageningen, Netherlands) [40]. To identify the pair-wise genetic relationships between the 96 accessions, a genetic distance matrix was calculated with GENALEX 6.4 [39]. Principal component analysis (PCA) based on genotype data of SSR markers was performed using GENALEX 6.4.

A Neighbor-Joining (NJ) tree based on Nei’s genetic distance [41] was also drawn in MEGA version 6.06 (Temple University, Philadelphia, PA, USA) [42]. Additionally, the population structure was inferred using the Bayesian clustering method implemented in the program STRUCTURE 2.2 (Stanford University, Stanford, CA, USA) [43]. The software was run with the admixture model and correlated allele frequencies. Five runs were performed for each *k* (1 to 10) representing the number of clusters considered. The burn-in number and iterations for each run were both set to 100,000 and the true *k* was determined according to the method described by [44]. Sesame accessions with membership probabilities ≥0.60 were assigned to the corresponding subgroup and accessions with membership probabilities <0.60 were assigned to a mixed subgroup. All accessions were mapped according to their geographical coordinates with ArcGIS software version 9.3 (Esri, Redlands, CA, USA). The geographical coordinates were obtained from the China National Genebank and for some accessions from Africa the originating country geographical coordinates were assigned.

## 3. Results

### 3.1. Assessment of the Intra-Accession Variability

A pre-test for assessing the variability within 24 accessions from West Africa with a single plant DNA extraction approach was performed. Out of the 35 markers used, only the marker ZMM1522 was polymorphic (2.86%), with two individuals among the 12 from the accession K1712 exhibiting different alleles. This result suggests that the genotyped accessions were relatively homozygous. 

Two markers including the marker ZMM1522 from the 35 markers were excluded and the 33 marker-genotype combinations that displayed clear bands were retained to analyze the bulked samples of the 96 accessions.

### 3.2. SSR Polymorphism in the Sesame Accessions

Thirty-three polymorphic SSRs were used to assess the genetic diversity in a sesame panel, with at least two markers per linkage group. A total of 137 alleles among the 96 sesame accessions were observed (Table S2). Number of alleles observed ranged from two (ZMM294, ZMM2202, ZMM2313, ZMM2321, ZMM2356, ZMM2734, ZMM2738) to 10 (ZMM1762), with an average of 4.15 alleles per locus. Major allele frequency (MAF) average was 0.59 and PIC average was 0.45 (Table 2).

### 3.3. Allele Variation among Geographical Regions

Diversity indices varied greatly between the six geographical regions. Among them, the highest MAF (0.7) was observed in North Africa (NAf), whereas quite similar MAFs were found in the five other groups (Table 2). The analysis of allelic patterns across the geographical regions revealed that accessions from West Africa (WAf) had the largest allele number (4.909). Africa showed a slightly higher mean number of alleles than Asia (5.2 *vs.* 4.8). Ne values ranged from 1.265 (West Asia) to 1.458 (South Asia). Values of He also varied among the different groups and ranged between 0.343 (North Africa) and 0.598 (South Asia). Asia showed relatively higher He values than Africa. The Shannon’s Information index (I) values ranged from 0.209 (West Asia) to 0.414 (South Asia).

Out of the 137 alleles, 49 (35.77%) were specific to geographical origin. West Africa and East Asia exhibited the highest private allele numbers (17 and 8, respectively), but no private alleles were found in West Asia, which might be due to the low sample number from this area. In general, the data showed that Africa harbored more private alleles (30) than Asia (21).

South Asia showed the highest PIC value (0.5421), whereas North Africa showed the lowest PIC value (0.2828). Generally, accessions from Asia displayed relatively higher values for He, I, and PIC than those from Africa.

### 3.4. Pattern of Genetic Diversity and Phylogenetic Relationships

**Figure 2:**
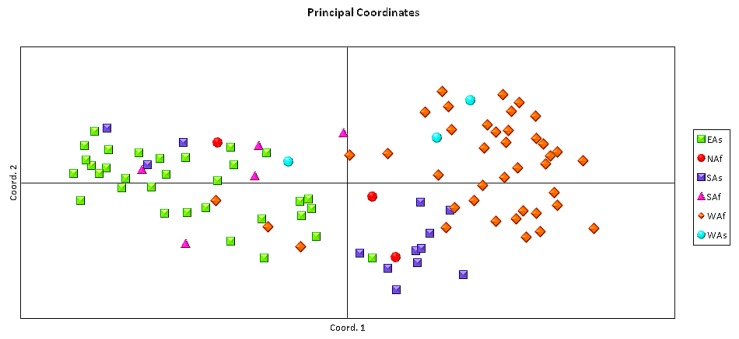
Principal component analysis of the 96 sesame accessions collected from six geographical regions. Each symbol represents one variety from one of the six studied regions.

The genetic distance matrix generated by GENALEX 6.4 was used for Principal Component Analysis of the 96 sesame accessions (Figure 2). The first and second axis, respectively, explained 36.40% and 18.22% of the variance within the molecular data. Populations from West Africa, South Asia, and East Asia were clearly distinguished by PCA analysis (Figure 2).

### 3.5. Analysis of Molecular Variance

The AMOVA results indicate that 44.66% of the total molecular variation in sesame accessions used in the study was partitioned among geographical groups, and 55.34% was attributed to differentiation within geographical groups (Table 3). In terms of continents, 34.95% of the total molecular variation observed was due to differentiation between Asia and Africa, whereas the rest (65.05%) was due to variance within continents (Table 4).

### 3.6. Population Structure

**Figure 4:**
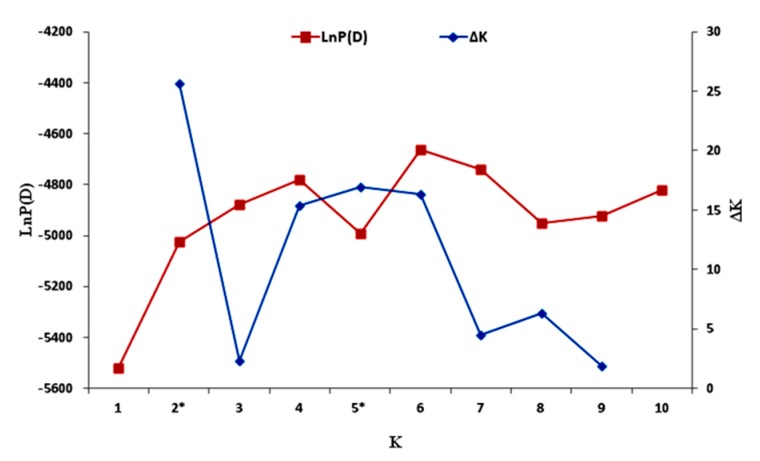
Estimated LnP(D) and ∆*k* of the 96 sesame accessions over five runs for each *K* value. * Significant *K* values identified.

The model-based approach implemented in STRUCTURE was performed to examine the relatedness among the 96 sesame accessions, using the genotypic data for 33 polymorphic SSRs. To facilitate the determination of the exact *k* value corresponding to the genetic groups, the *ad hoc* quantity Δ*k* was used. The highest value of Δ*k* for the 96 sesame accessions was for *k* = 2 (Figure 4).

**Figure 5:**
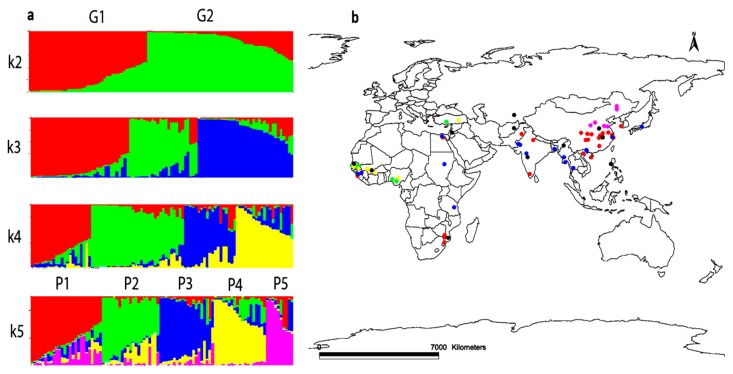
(**a**) Estimated population structure of sesame accessions assessed by STRUCTURE. Each individual is represented by a thin vertical bar, partitioned into up to *k* colored segments; (**b**) mapping the 96 sesame accessions based on their geographical regions. The different colors assigned represent the different groups inferred by STRUCTURE analysis. P1 = Red, P2 = Green, P3 = Blue, P4 = Yellow, P5 = Pink, Pmixed = Black.

The results indicated that all the accessions could be classified into two groups, designated as G1 and G2 (Figure 5a). Of the total accessions, 93.75% showed values of probability of membership higher than 0.6 and were therefore classified as members of a particular group, whereas six—representing only 6.25% of accessions—were classified as admixtures with degrees of membership (probability of membership <0.6) shared among the two genetic groups. G1 gathered together 39 accessions, including 31 from Asia, and G2 included in total 51 accessions, with 42 from Africa. The six accessions assigned to the admixed group include an accession from North Africa, three accessions from South Asia, and two accessions from East Asia (Table S3). It was thus suggested that based on genetic difference, the two major groups might be related to the two continents (Africa and Asia).

In order to better understand the geographical differentiation based on the defined geographical regions, the main groups were further subdivided into five subpopulations (P1–P5) according to STRUCTURE with *k* = 5 the highest after *k* = 2 (Figure 5a). It was observed that two subpopulations, P1 and P5, included most of the accessions from East Asia, whereas the other groups had accessions mainly from South Asia. Most of the West African accessions clustered in subpopulations P2 and P4, while the rest of the accessions from North Africa, Southeastern Africa, and West Asia were clustered among the five subpopulations with no clear pattern. These results might be influenced by the relatively small number of accessions from each of these three regions. Eleven accessions (11.45%) including eight from Asia, were assigned to the admixed population (Pmixed). The classification derived from the STRUCTURE analysis enabled the mapping of all accessions based on their geographical coordinates and their related subpopulations (Figure 5b). Subpopulation P5 was found to be clustered only in North East China, while subpopulation P1 was mostly found to cluster in South East China with some accessions from India, Guinea, Mozambique, and Egypt. Subpopulation P3 was observed to be distributed throughout most South Asian countries and some African countries such as Sudan, Tanzania, Guinea, and Egypt. Both P2 and P4 include accessions mostly from West Africa and Turkey.

## 4. Discussion

This study analyzed the genetic diversity and population structure of cultivated sesame accessions from Africa and Asia using 33 SSR markers. SSRs have widely been used in genetic diversity and evolutionary analysis in many crops because of their low cost, high polymorphism information content, reproducibility, co-dominant nature, and complete genome coverage [45,46]. The markers used in this study covered the whole genome and can provide a more comprehensive relationship analysis of the samples [33]. The number of alleles detected and the average values of He and PIC are higher than those reported by [32] and lower than reports of [33], where the authors used 150 and 33 worldwide sesame accessions genotyped with 16 and 216 SSR markers, respectively. The differences observed with other studies might be due to the use of different accessions, sampling approaches (bulk *vs.* individuals), and the number of SSR markers. In addition, the bulked sampling approach used in this study may have the tendency to influence the estimates of the diversity indices compared to the individual sampling approach, which is more informative. The genetic diversity observed in African accessions was lower than Asian accessions as shown by He, I PIC, and AMOVA results. These findings are in agreement with the low genetic diversity in African materials previously reported by [25]. In another study [9], sesame accessions from Asia were grouped into four geographical areas but all displayed higher genetic diversity than accessions from Africa. In general, the geographical focus of the higher genetic diversity of a species usually reveals its domestication center [47,48,49].

The findings from our study are in agreement with the earlier reports of sesame domestication from India [13,21] in the Asian continent.

It was observed in the study that accessions from three regions, namely East Asia, South Asia, and West Africa, had the highest diversity indices, suggesting that these regions contained more genetic diversity of sesame than the other geographical regions. Previous works [25,26] have also reported high genetic diversity of sesame accessions from South Asia and East Asia. However, the southeastern region of Africa was expected to contain more genetic diversity due to its long history of sesame cultivation [9,50,51]. The current study was likely to be influenced by the small sample size from this origin. There is therefore the need to collect more germplasm from Southeastern Africa to confirm whether the genetic diversity of sesame in this region is high or not. Although several genetic diversity studies of worldwide germplasm for sesame have been reported [6,9,25,32], to the best of our knowledge this is the first report on the genetic diversity of a large set of sesame accessions from West Africa, which was largely unexplored. The five subpopulations observed in the current study using both phylogenetic and STRUCTURE analyses are higher than in the report of Cho et al. [32], who found in total three subpopulations including two subpopulations of Korean accessions and one subpopulation comprising worldwide accessions. The higher number of subpopulations observed in the current study might be due to the use of more diverse and representative sesame accessions. Very few studies found a correlation between molecular marker patterns and the geographical origins of sesame, as observed with the accessions from South Asia, East Asia, and West Africa [33,52].

Earlier works in rice and sorghum where the researchers used 1794 worldwide accessions of rice and 3367 accessions of sorghum showed some level of correlation between geographical origins and SSR patterns [53,54].

We observed a close genetic relationship between accessions from East Africa, North Africa, and Guinea in West Africa to the accessions from Asia in the study. This close genetic relationship observed might be due to the introduction of sesame into many countries and material exchange from widely separated locations [28]. For instance, the similarity of Turkish and some West African materials could be explained by the exchange of sesame seeds through the research programs financed by IAEA (International Atomic Energy Agency) for breeding improved sesame cultivars. Moreover, the exchange of plant materials between Asia and East Africa dates back to a long time ago and is still occurring [55], with a steady increase in annual exportation of raw sesame seeds mainly for industrial applications but also for research purposes to China, India, Japan, and other countries. The possibility of crossover events between materials from different locations grown in the same area is high, knowing that cross-pollination in sesame has been reported to occur at a frequency between 5% and 60% [33]. This crossing could explain the similarity of accessions from the eastern part of Africa and Asia. Similar patterns have also been observed by other researchers [9,28,32].

According to [13], sesame was originally domesticated in South India and spread into different areas. In this study, a high proportion of private alleles were observed within each geographical region, with 44% of the variation among accessions being attributed to variation among geographical regions. This suggests that although geographically proximate subpopulations are genetically more similar than distant ones, differentiation is occurring in each population independently. It is therefore clear that the set of accessions used in this study included diverse accessions and could prove to be a valuable gene pool for allele mining and association mapping for future improvement of the sesame crop.

Finally, contrary to the conclusions of [9], based on the high variation detected within West African, East Asian, and South Asian accessions and their geographical differentiation, the diversity available to breeding programs can be maximized by selecting genotypes from these geographical origins.

A few previous studies have compared sesame’s genetic diversity between Asia and Africa, but in those studies the authors did not include a representative set of African materials compared to the Asian samples included. Our results fine-tune the previous knowledge about sesame diversity in both continents using a set of representative accessions. Large sesame germplasm collections are available in the gene banks of many countries such as the United States, South Korea, China, and India [14,34]. However, few sesame germplasm accessions from West Africa have been collected in these gene banks. From this study, there is an indication that new geographically isolated gene pools are evolving in West Africa. Future germplasm collections should focus on West African accessions, which could be exploited in breeding programs mainly oriented towards drought resistance. Moreover, a comparative analysis of the oil content and quality of a large set of sesame from both continents needs to be conducted so as to enable better selection of accessions for breeding purposes.

## Figures and Tables

**Figure 3:**
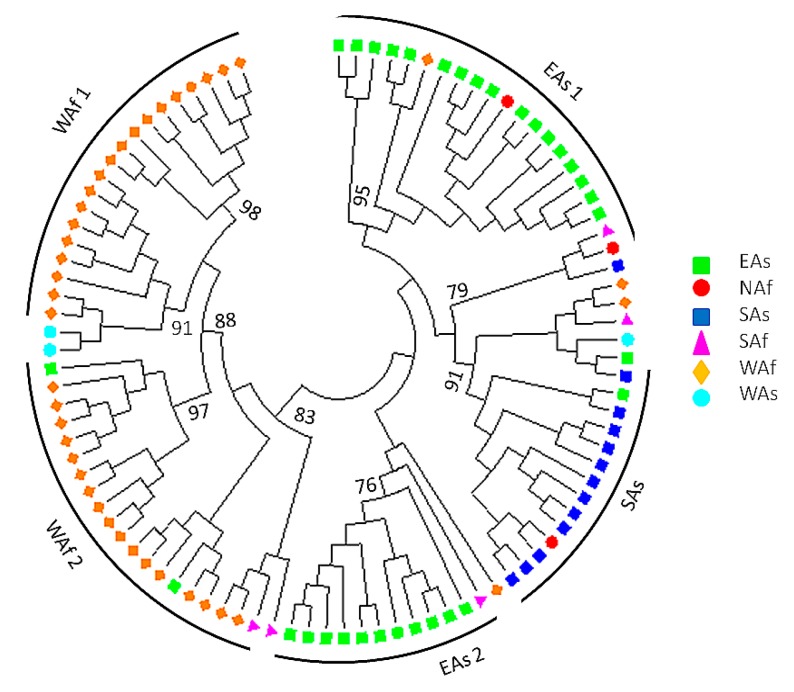
NJ tree showing the genetic relationships among the 96 sesame accessions. Bootstrap values >75 are shown.

**Table 1:** Summary of accessions used in the study.

Continents	Geographical Regions	Number of Countries	Number of Accessions
Asia	West Asia (WAs)	2	3
	East Asia (EAs)	5	32
	South Asia (SAs)	7	13
	Total	14	48
Africa	West Africa (WAf)	5	40
	North Africa (NAf)	2	3
	Southeast Africa (SAf)	2	5
	Total	9	48

**Table 2:** Summary of the genetic diversity of the sesame accessions based on their different geographical regions.

Groups	N	MAF	Na	Ne	He	Ho	I	PIC	Np
**Regions**									
**East Asia**	32	0.558 ± 0.138	4.030 ± 2.038	1.439 ± 0.355	0.565 ± 0.130	0	0.396 ± 0.250	0.501 ± 0.146	8
**West Asia**	3	0.585 ± 0.186	2.242 ± 0.560	1.265 ± 0.382	0.484 ± 0.161	0	0.209 ± 0.301	0.399 ± 0.153	0
**South Asia**	13	0.505 ± 0.176	3.848 ± 1.543	1.458 ± 0.349	0.598 ± 0.182	0	0.414 ± 0.240	0.542 ± 0.185	5
**North Africa**	3	0.707 ± 0.246	1.878 ± 0.739	1.385 ± 0.408	0.343 ± 0.261	0.006	0.303 ± 0.320	0.282 ± 0.224	5
**Southeastern Africa**	5	0.581 ± 0.214	2.787 ± 1.139	1.357 ± 0.377	0.509 ± 0.210	0	0.305 ± 0.297	0.445 ± 0.209	4
**West Africa**	40	0.578 ± 0.178	4.909 ± 2.350	1.379 ± 0.362	0.550 ± 0.179	0.002	0.346 ± 0.263	0.502 ± 0.178	17
**Continents**									
**Africa**	48	0.565 ± 0.184	5.242 ± 2.739	1.407 ± 0.348	0.559 ± 0.181	0.003	0.382 ± 0.235	0.510 ± 0.181	30
**Asia**	48	0.513 ± 0.145	4.848 ± 2.575	1.4712 ± 0.348	0.604 ± 0.134	0	0.430 ± 0.221	0.543 ± 0.151	21

These statistics are means across the 33 loci.

**Table 3:** AMOVA between the geographical regions.

Source	df	SS	MS	Est. Var.	%Tv	*p*
Among geographical regions	2	1103.034	551.517	20.135	44.66	0.001
Within geographical regions	82	2246.107	27.391	24.95	55.34	
Total	84	3349.141		45.085	100	

df = degree of freedom, SS = Sum of Square, MS = Mean Square, Est. Var. = Estimate Variance, %Tv = Percentage of total variation.

**Table 4:** AMOVA between the continents.

Source	df	SS	MS	Est. Var.	%Tv	*p*
Among continents	1	615.726	615.726	18.189	34.952	0.001
Within continents	94	3182.003	33.851	33.851	65.048	
Total	95	3797.729		41.999	100	

df = degree of freedom, SS = Sum of Square, MS = Mean Square, Est. Var. = Estimate Variance, %Tv = Percentage of total variation.

## Supplementary Materials

The following are available online at www.mdpi.com/2073-4425/7/4/14/s1. Table S1: Origin and summary phenotype data of the 96 accessions used in the study; Table S2: Characteristics of the 33 SSR markers used in this study; Table S3: Geographical coordinates and group, subpopulation, and probability of each accession inferred by STRUCTURE.
