# Supplementary material for: Analysis of Genetic Diversity and Population Structure of Sesame Accessions from Africa and Asia as Major Centers of Its Cultivation
Source: Genes (Basel). 2016 Apr 12;7(4):14. doi: 10.3390/genes7040014 (PMC4846844; doi:10.3390/genes7040014)
Supplement: Supplementary file 1 [file genes-07-00014-s001.docx]

Supplementary Materials: Analysis of Genetic Diversity and Population Structure of Sesame Accessions from Africa and Asia as Major Centres of Its Cultivation

Komivi Dossa, Xin Wei, Yanxin Zhang, Daniel Fonceka, Wenjuan Yang, Diaga Diouf, Boshou Liao, Ndiaga Cissé and Xiurong Zhang

**Table S1.** Origin and summary phenotype data of the 96 accessions used in the study.

| **Code** | **Origin** | **Geographical Regions** | **Seed Color** | **Breeding Status** | **Plant Type** | **Capsule Number per Axil** | **Capsule Edge Number** | **Stalk Pubescence Density** | **Corolla Color** |
| --- | --- | --- | --- | --- | --- | --- | --- | --- | --- |
| G801 | China | East Asia | light brown | Landrace | unbranched | 3 | 4/6 | few | pink |
| G802 | China | East Asia | brown | Landrace | unbranched | 3 | 4 | few | pink |
| G803 | China | East Asia | white | Modern cultivar | unbranched | 3 | 4/6/8 | much | purple |
| G804 | China | East Asia | brown | Landrace | branched | 1 | 4 | much | light |
| G806 | China | East Asia | white | Landrace | branched | 1 | 4/6 | much | light |
| G807 | China | East Asia | white | Landrace | branched | 1 | 4 | few | light |
| k1712 | Senegal | West Africa | white | Landrace | unbranched | NA | NA | few | purple |
| k1713 | Senegal | West Africa | white | Landrace | unbranched | 2 | 4 | few | pink |
| k1714 | Senegal | West Africa | white | Landrace | unbranched | 2 | 4 | few | purple |
| k1715 | Senegal | West Africa | white | Landrace | unbranched | 2 | NA | much | NA |
| k1716 | Senegal | West Africa | white | Landrace | unbranched | 2 | 4 | few | pink |
| k1717 | Senegal | West Africa | white | Landrace | unbranched | 2 | 4 | few | light |
| G808 | China | East Asia | white | Landrace | branched | 1 | 4 | medial | light |
| G809 | China | East Asia | white | Landrace | branched | 1 | 6/8 | much | pink |
| G810 | China | East Asia | white | Landrace | unbranched | 3 | 4 | much | pink |
| G811 | China | East Asia | white | Landrace | unbranched | 3 | 4/6/8 | much | pink |
| G812 | China | East Asia | white | Landrace | branched | 3 | 4 | much | pink |
| G813 | China | East Asia | black | Landrace | unbranched | 1 | 8 | medial | pink |
| G814 | China | East Asia | white | Landrace | unbranched | 3 | 8 | few | light |
| G818 | China | East Asia | black | Landrace | unbranched | 3 | 6/8 | much | pink |
| G819 | China | East Asia | white | Landrace | branched | 1 | 4/6 | much | pink |
| G845 | China | East Asia | white | Landrace | unbranched | 1 | 6/8 | medial | pink |
| G854 | China | East Asia | white | Landrace | unbranched | 3 | 4 | medial | pink |
| G858 | China | East Asia | light brown | Landrace | branched | 1 | 4/6 | medial | pink |
| G859 | China | East Asia | white | Landrace | branched | 3 | 4 | medial | NA |
| G419 | Uzbekistan | South Asia | light brown | Landrace | branched | 1 | 4 | medial | pink |
| G840 | China | East Asia | white | Landrace | branched | 3 | 4/6/8 | medial | pink |
| G843 | China | East Asia | white | Landrace | branched | 3 | 4 | medial | light |
| G820 | China | East Asia | brown | Landrace | branched | 3 | 4 | much | light |
| G821 | China | East Asia | black | Landrace | branched | 1 | 6/8 | much | NA |
| G822 | China | East Asia | white | Landrace | branched | 1 | 6/8 | medial | light |
| G825 | China | East Asia | white | Landrace | unbranched | 1 | 4 | much | pink |
| G874 | Vietnam | South Asia | white | Landrace | unbranched | 3 | 4 | much | pink |
| G876 | Bangladesh | South Asia | brown | Landrace | branched | 1 | 4 | much | pink |
| G593 | Guinea | West Asia | white | Landrace | branched | 1 | 4 | much | light |
| G595 | Guinea | West Asia | brown | Landrace | branched | 1 | 4 | much | pink |
| G623 | United Arab Emirates | West asia | brown | Landrace | branched | 1 | 4 | much | pink |
| G877 | India | South Asia | white | Landrace | branched | 3 | 4 | few | pink |
| G626 | Egypt | North Africa | white | Landrace | branched | 3 | 4 | medial | pink |
| k1697 | Mali | West Africa | white | Landrace | unbranched | 2 | 4 | few | light |
| k1698 | Mali | West Africa | white | Landrace | unbranched | 2 | 4 | few | light |
| k1699 | Mali | West Africa | white | Landrace | unbranched | 2 | 4 | few | purple |
| k1700 | Mali | West Africa | white | Landrace | unbranched | 2 | 4 | few | purple |
| k1701 | Senegal | West Africa | white | Landrace | unbranched | 2 | 4 | few | light |
| k1702 | Senegal | West Africa | white | Landrace | unbranched | NA | NA | much | NA |
| k1703 | Senegal | West Africa | brown | Landrace | unbranched | 2 | 4 | few | light |
| k1704 | Turkey | West Asia | brown | Landrace | unbranched | NA | NA | few | NA |
| k1705 | Turkey | West Asia | brown | Landrace | unbranched | 2 | 4 | few | pink |
| k1706 | Senegal | West Africa | brown | Landrace | unbranched | NA | NA | few | light |
| k1707 | Senegal | West Africa | brown | Landrace | branched | 2 | 4 | few | purple |
| k1708 | Senegal | West Africa | white | Modern cultivar | unbranched | 2 | 4 | few | purple |
| k1709 | Senegal | West Africa | white | Modern cultivar | unbranched | 2 | 4 | few | light |
| k1710 | Senegal | West Africa | white | Landrace | unbranched | NA | NA | few | NA |
| k1711 | Senegal | West Africa | white | Landrace | unbranched | NA | NA | few | light |
| k1718 | Senegal | West Africa | white | Landrace | unbranched | 2 | 4 | few | purple |
| G519 | India | South Asia | black | Landrace | unbranched | 3 | 4 | few | pink |
| G522 | India | South Asia | black | Landrace | branched | 1 | 4 | few | pink |
| G524 | Thailand | East Asia | light brown | Landrace | branched | 1 | 4 | few | pink |
| G527 | India | South Asia | white | Landrace | unbranched | 1 | 6/8 | much | pink |
| G870 | India | South Asia | light brown | Landrace | unbranched | 1 | NA | much | purple |
| G871 | India | South Asia | brown | Landrace | branched | 3 | 4 | much | light |
| G543 | Egypt | North Africa | brown | Landrace | branched | 1 | 4 | much | pink |
| G551 | Israel | West Asia | white | Landrace | unbranched | 1 | 4 | much | pink |
| G553 | Pakistan | South Asia | light brown | Landrace | branched | 1 | 4 | much | pink |
| k1719 | Senegal | West Africa | white | Landrace | unbranched | 2 | 4 | few | purple |
| k1720 | Senegal | West Africa | white | Landrace | unbranched | 2 | 4 | few | purple |
| k1721 | Senegal | West Africa | white | Landrace | unbranched | 2 | 4 | few | purple |
| k1722 | Senegal | West Africa | white | Landrace | unbranched | 2 | 4 | few | light |
| k1723 | Senegal | West Africa | white | Landrace | unbranched | 2 | 4 | few | light |
| k1724 | Senegal | West Africa | white | Landrace | branched | 2 | 4 | few | light |
| k1725 | Senegal | West Africa | white | Landrace | unbranched | 2 | 4 | few | purple |
| k1726 | Senegal | West Africa | brown | Landrace | branched | 2 | 4 | few | pink |
| k1727 | Senegal | West Africa | brown | Landrace | branched | 2 | 4 | few | light |
| k1728 | Senegal | West Africa | brown | Landrace | unbranched | NA | NA | few | NA |
| HNT003 | Nigeria | West Africa | white | Landrace | NA | NA | NA | NA | NA |
| G468 | Mozambique | Southeastern Africa | white | Landrace | unbranched | 1 | 6/8/10 | much | light |
| G469 | Mozambique | Southeastern Africa | white | Landrace | branched | 3 | 4 | much | pink |
| G867 | South Korea | East Asia | white | Landrace | unbranched | 3 | 4 | much | pink |
| G509 | Philippines | East Asia | brown | Landrace | branched | 1 | 4 | few | pink |
| G868 | Myanmar | South Asia | black | Landrace | branched | 3 | 4 | few | pink |
| G826 | China | East Asia | light brown | Landrace | branched | 1 | 4 | much | light |
| G837 | China | East Asia | light brown | Landrace | branched | 1 | 4 | few | light |
| G839 | China | East Asia | white | Landrace | branched | 1 | 6/8 | medial | pink |
| k1729 | Senegal | West Africa | brown | Landrace | unbranched | NA | NA | few | NA |
| k1730 | Senegal | West Africa | brown | Landrace | unbranched | 2 | 4 | much | purple |
| k1731 | Senegal | West Africa | brown | Landrace | branched | NA | NA | NA | NA |
| k1777 | Burkina Faso | West Africa | white | Modern cultivar | unbranched | 4 | 4 | few | purple |
| HNT001 | Nigeria | West Africa | white | Landrace | NA | NA | NA | NA | NA |
| G425 | Sudan | Southeastern Africa | black | Landrace | branched | 1 | 4 | medial | pink |
| G862 | Myanmar | South Asia | brown | Landrace | branched | 1 | 4 | medial | pink |
| G428 | Tanzania | Southeastern Africa | white | Landrace | branched | 1 | 4 | medial | light |
| G436 | Guinea | West Asia | white | Landrace | branched | 3 | 4 | few | pink |
| G865 | Mozambique | Southeastern Africa | light brown | Landrace | unbranched | 3 | 4 | few | light |
| HNT002 | Nigeria | West Africa | white | Modern cultivar | NA | NA | NA | NA | NA |
| G452 | Mozambique | Southeastern Africa | light brown | Landrace | unbranched | 3 | 4 | medial | pink |
| G458 | Japan | East Asia | brown | Landrace | unbranched | 3 | 4 | medial | pink |

**Table S2.** Characteristics of the 33 SSR markers used in this study obtained from work of Wei et al. (2014) *.

| **Primer** | **LG** | **Forward Primer Sequence** | **Reverse Primer Sequence** | **Start Position (bp)** | **End Position (bp)** | **Allele No.** | **He** | **Ho** | **MAF** | **PIC** |
| --- | --- | --- | --- | --- | --- | --- | --- | --- | --- | --- |
| ZMM294 | 13 | GGCCAACCCTTTTCAGATTT | GGGCTTCACAACACAAGACA | 3908796 | 3,908,815 | 2 | 0.56 | 0.03 | 0.56 | 0.5 |
| ZMM334 | 16 | AATTGGACTCCGGCTAGGAT | CGCCCTCATCCTTACAATCT | 4181952 | 4,181,971 | 5 | 0.38 | 0 | 0.75 | 0.6 |
| ZMM425 | 14 | CAAGTCGCCATCACACTCAT | TCGAGTTGGAATGCAACAAA | 22169 | 22,188 | 5 | 0.46 | 0 | 0.65 | 0.7 |
| ZMM689 | 11 | CGCTTGAATTAATTGCATCTACC | CCAAGTGAACATAGAAATCTGCC | 4249715 | 4,249,737 | 4 | 0.71 | 0 | 0.41 | 0.6 |
| ZMM715 | 16 | CCCCTCTCAAATAAGCCCTC | AGGAAGGAGGGTGTCCCTAA | 4962006 | 4,962,025 | 5 | 0.61 | 0 | 0.48 | 0.8 |
| ZMM734 | 4 | CCAGACCCAAACCCAATAGA | TGCATTTAAGGCTGTGCAAC | 16185910 | 16,185,929 | 3 | 0.62 | 0 | 0.48 | 0.7 |
| ZMM987 | 1 | CTTTGATTGGGCCACCCTA | TGTTTGTTCTTCTTCCCCCA | 5516846 | 5,516,865 | 9 | 0.62 | 0 | 0.52 | 0.9 |
| ZMM1194 | 8 | TTCCAATTCTACAAGCGCAG | CCGATCAAAACTAGTATGGCAA | 4072488 | 4,072,509 | 3 | 0.67 | 0 | 0.44 | 0.6 |
| ZMM1202 | 9 | AATTAAAATTGGCCCAAGCC | GGCCAAGTGGAAATTTGAAG | 10093205 | 10,093,224 | 4 | 0.78 | 0 | 0.38 | 0.7 |
| ZMM1303 | 14 | TCCCAATCAGTTAGGTCGAG | TTAAGCTTAGGGGTCGGGTT | 1015459 | 1,015,478 | 4 | 0.81 | 0 | 0.23 | 0.5 |
| ZMM1383 | 8 | TCATTAACCCATCATTGCGA | TGCTCACACATAACAGTTGGG | 391893 | 391,913 | 4 | 0.49 | 0.01 | 0.57 | 0.4 |
| ZMM1415 | 1 | TGTTGTTTGACCGTCTTCCA | TCGGGCTAGAAACCAACAGT | 15890198 | 15,890,217 | 5 | 0.56 | 0 | 0.48 | 0.7 |
| ZMM1648 | 15 | TCCTGAATTCAAACGCATTG | TCCTAAACCCTCTGCACCAC | 6841254 | 6,841,273 | 7 | 0.59 | 0 | 0.59 | 0.7 |
| ZMM1700 | 5 | CATTAACACCATTACGCAAACA | TTTGGCAAAACTGCAATGAA | 16338016 | 16,338,037 | 6 | 0.8 | 0 | 0.29 | 0.8 |
| ZMM1762 | 6 | TGCAAGGACAACCAAAATCA | TGCACTGCATTGTCTCCTTT | 14685839 | 14,685,858 | 10 | 0.68 | 0 | 0.45 | 0.8 |
| ZMM1816 | 11 | CCAGCTCTATTGTGCGTTGA | CACTGCTTTCTCTGAAAGGCT | 10438707 | 10,438,727 | 3 | 0.5 | 0 | 0.6 | 0.5 |
| ZMM2202 | 6 | TCAGGAAGAAAGAATTGCTGC | CAACCCAACCATCCTGACTC | 8302871 | 8,302,891 | 2 | 0.72 | 0 | 0.36 | 0.4 |
| ZMM2218 | 3 | GATGGGGAAAGAGATTGGGT | ATTGAATCGACGTAATTTATCCTT | 18119689 | 18,119,712 | 5 | 0.49 | 0 | 0.57 | 0.6 |
| ZMM2254 | 1 | GCTTCCACCTAGCTCGGTTAT | CCAGCAATCATGTCTGCTTAAT | 13060305 | 13,060,326 | 3 | 0.43 | 0 | 0.72 | 0.5 |
| ZMM2283 | 12 | CGCCTTTCTCCTCCTTATCC | CATTCAGTCTTACGTCCAAATTTCT | 2859857 | 2,859,881 | 5 | 0.25 | 0 | 0.85 | 0.6 |
| ZMM2285 | 12 | ACTGCACCCTCTGCATTTTT | GCACGTGTGGGGTACCTTTA | 3085980 | 3,085,999 | 4 | 0.57 | 0 | 0.6 | 0.5 |
| ZMM2313 | 3 | TCAAAGTGTACCACAAAACGC | TCTTTCTCTCTCAATCATTTGTTTATG | 7203135 | 7,203,161 | 2 | 0.54 | 0 | 0.6 | 0.4 |
| ZMM2321 | 15 | CAACACCACCAACGCATATC | AGCAACGATTCACGACATTG | 3291876 | 3,291,895 | 2 | 0.78 | 0 | 0.28 | 0.3 |
| ZMM2356 | 13 | GGGGTGAGTATTTCGGGAGT | TCCATGCATCTTTTACACTGAA | 1568034 | 1,568,055 | 2 | 0.67 | 0 | 0.42 | 0.4 |
| ZMM2494 | 9 | GTCCGTCAACTCGATCACCT | TTCAACCAAACCCCATCATT | 92576 | 92,595 | 4 | 0.54 | 0 | 0.63 | 0.5 |
| ZMM2562 | 4 | GCCATTTCTTCATTTGGTGC | GCATTTCAATTTTTCACCCC | 16089230 | 16,089,249 | 3 | 0.61 | 0 | 0.56 | 0.4 |
| ZMM2734 | 2 | TGGACAAAGACACAATCACACA | TTGAATTTCGATCTTTCCATCA | 7565502 | 7,565,523 | 2 | 0.73 | 0 | 0.38 | 0.2 |
| ZMM2738 | 2 | CTGATGCAAAAACTGCCAAA | ACCGCACTCAAAGGTTCAAT | 7595028 | 7,595,047 | 2 | 0.73 | 0 | 0.4 | 0.4 |
| ZMM2793 | 7 | TGGTTCATACATCCTCTTTTTGG | CATAATTGATGCCTAAATTTCTCC | 10103745 | 10,103,768 | 3 | 0.79 | 0 | 0.26 | 0.5 |
| ZMM2818 | 5 | CGTGTGCCCAATATTTGAGTT | TCAACCTCCTCCCTACACAA | 12108011 | 12,108,031 | 4 | 0.43 | 0 | 0.74 | 0.6 |
| ZMM2877 | 10 | AAAATCCTCTTTTTCCGACGA | GATTTTGACACCTTTGCCTGA | 4751627 | 4,751,647 | 4 | 0.87 | 0 | 0.2 | 0.5 |
| ZMM3037 | 10 | ACACATACGGACAGGCACAG | ATATAGCCAGTTTGGCTGCG | 938674 | 938,693 | 4 | 0.66 | 0 | 0.47 | 0.7 |
| ZMM3059 | 7 | TTCTGTCACCAAGAATTGCG | GTCAAAATTGAGGGTTGCGT | 10112764 | 10,112,783 | 7 | 0.74 | 0 | 0.35 | 0.8 |
|  | | | **Mean** |  |  | **4.15** | **0.62** | **0.0012** | **0.49** | **0.57** |
|  |  |  | **Max** |  |  | **10** | **0.87** | **0.03** | **0.85** | **0.9** |
|  |  |  | **Min** |  |  | **2** | **0.25** | **0** | **0.2** | **0.2** |

* See References section in the article.

**Table S3.** Geographical coordinates and Group, Subpopulation, Probability of each accession inferred by STRUCTURE.

| **Samples ID** | **Longitude (°)** | **Latitude (°)** | **Structure Groups** | **Structure Subpopulations** | **Probability** |
| --- | --- | --- | --- | --- | --- |
| G468 | 32.35 | 25.5 | G1 | P1 | 0.774 |
| G469 | 32.45 | 25.7 | G1 | P1 | 0.649 |
| G452 | 32.40 | 25.1 | G1 | P1 | 0.677 |
| G519 | 79.19 | 28.1 | G1 | P1 | 0.894 |
| G527 | 77.32 | 10.2 | G1 | P1 | 0.799 |
| G553 | 73.0 | 31.2 | G1 | P1 | 0.856 |
| G593 | −13.4 | 9.3 | G1 | P1 | 0.659 |
| G626 | 33.1 | 32.0 | G1 | P1 | 0.617 |
| G806 | 114.3 | 30.3 | G1 | P1 | 0.956 |
| G808 | 121.4 | 29.2 | G1 | P1 | 0.637 |
| G811 | 109.8 | 31.0 | G1 | P1 | 0.642 |
| G812 | 113.8 | 29.2 | G1 | P1 | 0.83 |
| G813 | 120.7 | 30.7 | G1 | P1 | 0.697 |
| G819 | 115.7 | 29.5 | G1 | P1 | 0.924 |
| G822 | 114.0 | 24.9 | G1 | P1 | 0.707 |
| G825 | 115.8 | 31.6 | G1 | P1 | 0.801 |
| G839 | 108.1 | 28.2 | G1 | P1 | 0.613 |
| G840 | 106.8 | 28.1 | G1 | P1 | 0.678 |
| G843 | 118.7 | 32.0 | G1 | P1 | 0.933 |
| G845 | 109.8 | 19.0 | Gmixed | P1 | 0.653 |
| G854 | 107.7 | 31.3 | G1 | P1 | 0.607 |
| G858 | 104.4 | 31.8 | G1 | P1 | 0.712 |
| G867 | 125.4 | 35.3 | G1 | P1 | 0.728 |
| G874 | 105.5 | 21.0 | G1 | P1 | 0.666 |
| k1701 | −16.1 | 14.5 | G2 | P2 | 0.647 |
| k1702 | −16.0 | 14.4 | G2 | P2 | 0.722 |
| k1705 | 33.4 | 37.6 | G2 | P2 | 0.604 |
| k1708 | −16.0 | 14.3 | G2 | P2 | 0.743 |
| k1710 | −16.0 | 14.1 | G2 | P2 | 0.884 |
| k1711 | −15.9 | 14.3 | G2 | P2 | 0.642 |
| k1714 | −15.8 | 14.1 | G2 | P2 | 0.838 |
| k1715 | −15.8 | 14.4 | G2 | P2 | 0.832 |
| k1717 | −15.8 | 14.5 | G2 | P2 | 0.935 |
| k1719 | −15.8 | 15.0 | G2 | P2 | 0.612 |
| k1720 | −15.6 | 14.2 | G2 | P2 | 0.69 |
| k1722 | −15.5 | 14.5 | G2 | P2 | 0.649 |
| k1723 | −15.4 | 14.4 | G2 | P2 | 0.936 |
| k1724 | −16.2 | 14.1 | G2 | P2 | 0.956 |
| k1725 | −16.1 | 14.5 | G2 | P2 | 0.68 |
| k1726 | −15.3 | 15.0 | G2 | P2 | 0.675 |
| k1727 | −15.0 | 14.2 | G2 | P2 | 0.773 |
| HNT002 | 5.0 | 7.4 | G2 | P2 | 0.607 |
| HNT003 | 7.1 | 6.4 | G2 | P2 | 0.63 |
| G425 | 32.4 | 15.4 | G2 | P3 | 0.911 |
| G428 | 37.5 | 7.1 | G2 | P3 | 0.948 |
| G436 | −13.4 | 9.3 | G1 | P3 | 0.648 |
| G458 | 136.5 | 35.1 | G1 | P3 | 0.726 |
| G522 | 75.5 | 21.0 | G2 | P3 | 0.82 |
| G524 | 100.3 | 13.4 | G2 | P3 | 0.661 |
| G543 | 31.1 | 30.0 | Gmixed | P3 | 0.617 |
| G595 | −13.4 | 9.4 | G1 | P3 | 0.738 |
| G814 | 120.8 | 29.5 | G2 | P3 | 0.774 |
| G818 | 115.8 | 29.6 | G2 | P3 | 0.697 |
| G820 | 107.3 | 22.4 | G1 | P3 | 0.659 |
| G821 | 107.2 | 22.8 | G2 | P3 | 0.887 |
| G862 | 96.0 | 16.4 | G2 | P3 | 0.92 |
| G868 | 95.3 | 19.0 | Gmixed | P3 | 0.84 |
| G871 | 71.3 | 26.1 | G2 | P3 | 0.855 |
| G876 | 90.2 | 23.4 | G2 | P3 | 0.924 |
| G877 | 72.2 | 25.5 | G2 | P3 | 0.846 |
| k1697 | −8.4 | 12.8 | G2 | P4 | 0.629 |
| k1698 | −7.4 | 12.7 | G2 | P4 | 0.829 |
| k1699 | −6.8 | 12.5 | G2 | P4 | 0.602 |
| k1703 | −12.8 | 13.2 | G2 | P4 | 0.686 |
| k1704 | 39.8 | 38.4 | G2 | P4 | 0.603 |
| k1706 | −15.6 | 14.3 | G2 | P4 | 0.948 |
| k1707 | −15.7 | 14.5 | G2 | P4 | 0.626 |
| k1709 | −15.8 | 14.3 | G2 | P4 | 0.958 |
| k1712 | −15.7 | 14.1 | G2 | P4 | 0.644 |
| k1713 | −16.2 | 15.1 | G2 | P4 | 0.901 |
| k1718 | −14.2 | 14.3 | G2 | P4 | 0.841 |
| k1721 | −15.3 | 14.8 | G2 | P4 | 0.943 |
| k1728 | −15.3 | 14.5 | G2 | P4 | 0.739 |
| k1729 | −15.2 | 14.4 | G2 | P4 | 0.897 |
| k1730 | −15.1 | 14.3 | G2 | P4 | 0.677 |
| k1731 | −15.0 | 14.2 | G2 | P4 | 0.78 |
| k1777 | −4.5 | 11.1 | G2 | P4 | 0.63 |
| HNT001 | 8.2 | 8.3 | G2 | P4 | 0.61 |
| G801 | 116.5 | 35.4 | G1 | P5 | 0.925 |
| G802 | 118.3 | 35.1 | G1 | P5 | 0.837 |
| G804 | 113.8 | 34.0 | G1 | P5 | 0.659 |
| G807 | 123.2 | 45.8 | G1 | P5 | 0.939 |
| G809 | 111.7 | 37.2 | G1 | P5 | 0.936 |
| G810 | 109.4 | 35.7 | G1 | P5 | 0.617 |
| G859 | 123.3 | 44.1 | G1 | P5 | 0.792 |
| G419 | 69.1 | 41.1 | Gmixed | Pmixed | 0.45 |
| G509 | 120.1 | 15.5 | G2 | Pmixed | 0.383 |
| G551 | 34.4 | 32.0 | G1 | Pmixed | 0.398 |
| G623 | 69.1 | 34.3 | Gmixed | Pmixed | 0.422 |
| G803 | 113.9 | 34.2 | G1 | Pmixed | 0.495 |
| G826 | 115.8 | 29.3 | G1 | Pmixed | 0.488 |
| G837 | 95.2 | 25.2 | Gmixed | Pmixed | 0.365 |
| G865 | 34.4 | −23.1 | G1 | Pmixed | 0.414 |
| G870 | 76.2 | 19.1 | G1 | Pmixed | 0.462 |
| k1700 | −5.8 | 12.1 | G2 | Pmixed | 0.488 |
| k1716 | −15.1 | 15.6 | G2 | Pmixed | 0.522 |
